# Folate gene polymorphisms *CBS* 844ins68 and *RFC1* A80G and risk of Down syndrome offspring in young Iranian women: A cross-sectional study

**DOI:** 10.18502/ijrm.v22i2.15709

**Published:** 2024-03-25

**Authors:** Neda Farajnezhad, Pegah Ghandil, Maryam Tahmasebi-Birgani, Javad Mohammadi-Asl

**Affiliations:** ^1^Department of Medical Genetics, School of Medicine, Ahvaz Jundishapur University of Medical Sciences, Ahvaz, Iran.; ^2^Cellular and Molecular Research Center, Medical Basic Sciences Research Institute, Ahvaz Jundishapur University of Medical Sciences, Ahvaz, Iran.; ^3^Noorgene Genetics Laboratory, Ahvaz, Iran.

**Keywords:** Down syndrome, Folic acid, Polymorphism, CBS, RFC1.

## Abstract

**Background:**

Cytogenetics and association studies showed that folate gene polymorphisms can increase the risk of chromosomal nondisjunction and aneuploidies. The folate-metabolizing gene polymorphisms in Down syndrome mothers (DSM) have been assessed in a variety of populations. Reduced folate carrier 1 (*RFC1*) and cystathionine beta-synthase (*CBS*) are key enzymes in folate metabolism.

**Objective:**

2 common polymorphisms, *CBS* 844ins68 and *RFC1* A80G, were analyzed to determine their probable risk for having Down syndrome (DS) babies in young mothers of Khuzestan province, Iran.

**Materials and Methods:**

This study was conducted on 100 mothers who had trisomy 21 DS children. 100 age- and ethnic-matched mothers with at least 2 healthy children and no history of abnormal pregnancies were considered as control. The samples were collected from all the mothers from June 2019 to April 2021. Genomic DNA was extracted from peripheral blood. The *CBS*-844ins68 and *RFC1*-A80G were genotyped using polymerase chain reaction-electrophoresis and restriction fragment length polymorphism, respectively.

**Results:**

The frequency of *RFC1* AG and GG genotypes in DSM was significantly higher than the control mothers (odds ratio [OR] of 2.38 and 3.07, respectively). The heterozygote genotype of *CBS* 844ins68 was significantly more prevalent among DSM than the control (OR: 2.419). The OR was significantly increased to 6.667 when the homozygote of both variants was found together.

**Conclusion:**

Studying polymorphisms possibly increases the susceptibility of having a DS child. However, ethnicity, nutrition, and epistatic interactions are considerable factors to be evaluated in future studies.

## 1. Introduction

One of the most prevalent chromosomal causes of intellectual disability has been connected to Down syndrome (DS) (OMIMI # 190685). The syndrome is characterized by full or partial trisomy of chromosome 21. The incidence is approximately 1 in 1000 newborns and varies among the different populations (1). Full trisomy 21 is mainly caused by chromosomal nondisjunction during maternal meiosis-I. Free trisomy 21 is a sporadic event- if recurrence occurs, the gonadal mosaicism and Robertsonian translocation should be checked. Advanced maternal age ( ≥ 35 yr) is a well-approved risk factor for DS. However, several women < 35 yr at conception are predisposed to chromosomal nondisjunction and DS babies (2).

Folate is a natural form of vitamin B9, playing a vital role in DNA synthesis and methylation. In 1999, irregular folate metabolism was suggested to increase the probability of chromosome 21 nondisjunction through impairing the methylation levels in the pericentromeric regions of chromosome 21 and triggering chromosomal nondisjunction and genomic instability, (3, 4). Various studies considered the association between maternal folate gene polymorphisms and increased risk of DS; however, the results are controversial and remains questionable whether these polymorphisms in the mother are causal in determining DS susceptibility in the offspring (4, 5).

Here, 2 of the well-known folate genes are considered. The folate transporter reduced folate carrier protein (*RFC1*) on chromosome 21, mediates folate uptake into the cells. The polymorphism A80G (rs1051266) is the most prevalent variant in the *RFC1* gene replacing the amino acid histidine with arginine in the exon-2 and decreasing the plasma levels of folate and homocysteine. A positive association between A80G and DS was documented in different populations (6). Cystathionine beta-synthase (*CBS*) is another vital enzyme in folate metabolism and catalyzes the conversion of homocysteine and serine to cystathionine. Polymorphism c.844ins68 is a frequent polymorphism of the *CBS* gene that results in an insertion of a 68-bp within exon 8 and duplicates the intron 7 acceptor splice site, which may lead to 2 *CBS* mRNA variants through alternative splicing. Previously published studies have documented that the *CBS* 844ins68 polymorphism may be related to DS (7).

The goal of this study was to see if polymorphisms *CBS* 844ins68 and *RFC1* A80G may be linked to an increased risk of having DS among young Iranian mothers and used as maternal risk factor for DS. Although several researches on the function of Hcy/folate metabolism in the etiology of DS has been published in different geographical regions and populations, bringing both supportive data and innovative understandings, no study has existed on these polymorphisms in the Iranian population. The genetic counseling of DS would benefit significantly if the *RFC1* A80G and *CBS* 844in68 polymorphisms could be associated to an increased risk of developing DS in females. Recent studies have suggested that DS children are more likely to be born from young mothers (8), we selected mothers whose age was under 30 yr at the time of pregnancy with DS babies. Moreover, we tried to reduce the effect of maternal age on the incidence of DS.

Therefore, in mothers of DS children, the syndrome may have arisen from something other than maternal age. To our knowledge, the present study is a basic study whose data can be useful in revealing the molecular mechanisms of the process of chromosome nondisjunction, and its goal is not to reach a diagnostic method and an alternative to conventional DS screening systems.

## 2. Materials and Methods

This cross-sectional study was conducted on 200 participants from June 2019 to April 2021 in Iran. Genomic DNA was extracted from the peripheral blood of 100 DS mothers (DSM) and 100 control mothers (CM). *CBS* 844ins68 was genotyped using polymerase chain reaction (PCR)-electrophoresis, while *RFC1* A80G was analyzed through restriction fragment length polymorphism (RFLP).

### Study population

To decrease the effect of maternal age, mothers older than 35 were excluded (n = 35) from this study. Then, 100 mothers, aged below 35 (n = 100), who had given birth to at least 1 child with DS, confirmed to have full trisomy 21 by karyotyping, were included in this study as DSM. Both mothers and fathers of children with DS having normal karyotype and carriers of translocation or iso chromosomes were excluded from this study. They were collected from June 2019 to April 2021 through medical genetics laboratories. 100 age- and ethnic-matched mothers whose children were not affected by DS were included in this project as CM. They had at least 2 healthy children and no history of miscarriage or abnormal pregnancies. All mothers belonged to the Khuzestan Province. The serum folate level of both DSM and CM were within the normal range. A questionnaire was used to collect data from mothers and all enrolled participants signed the informed consent.

### Genomic DNA extraction

Genomic DNA was extracted from 5 mL of peripheral blood lymphocytes using a DNA extraction kit (Kiagene, Iran) according to its user protocol. Extracted DNAs were stored at -20 C until use.

### Detection of polymorphisms

#### *CBS* 844ins68 genotyping

Since 844ins68 polymorphism is a 68-bp insertion in the *CBS* gene, genotyping was done using PCR and agarose gel electrophoresis. Specific forward and reverse primers were 5 ' -CGCCCTCTGCAGATCATTGG-3 ' and 5 ' -CCTTCCACCTCGTAGGTTGTC-3 ' , respectively, to amplify a 98 bp amplicon of the *CBS* gene in wild-type. The PCR reaction was prepared in the total volume of 25 μl containing 1 × reaction buffer, 0.25 mM of each dNTP, 2 pmol/l of each primer, 0.4 μg genomic DNA templates, and 1.5 U of Taq DNA polymerase. The PCR was set up in thermocycler as 5 min denaturation at 95 C for initial denaturation followed by 33 cycles of denaturation; 95 C (1 min), annealing; 62 C (45 sec), extension; 72 C (45 sec), final extension; 72 C (7 min). A negative control reaction was prepared in all PCR reactions. The 2% agarose gel electrophoresis was used to discriminate all possible genotypes. The expected electrophoretic pattern was a 98 bp fragment for the wild-type homozygous genotype (Ins-/-), 2 fragments of 98 bp and 166 bp for the heterozygous genotype (Ins+/-), and a fragment of 166 bp for *CBS* 844ins68 homozygous genotype (Ins+/+).

#### *RFC1* A80G genotyping

Polymorphism *RFC1* A80G genotype analyses were done using PCR-RFLP. Specific forward and reverse primers were 5 ' -AGTGTCACCTTCGTCCC-3 ' and 5 ' -GTTCTTGAAGTGCGCCCT-3 ' , respectively. The PCR was set up in a thermocycler as 5 min denaturation at 95 C for initial denaturation followed by 33 cycles of denaturation; 95 C (1 min), annealing; 53 C (45 sec), extension; 72 C (45 sec), final extension; 72 C (7 min). The PCR products were then subjected to digestion with C*fo*I (Sigma) endonuclease for 3 hr. The expected RFLP pattern were 3 fragments of 125, 68, and 37 bp in the presence of the 80G allele, while the 80A allele produced 2 pieces of 162 and 68 bp.

### Sanger DNA sequencing

The amplicon sequence was then read using Big Dye Terminators (Applied Bio systems ${\;}^{\text{TM}}$ , Genetic Analyzer; Applied Biosystems, Foster City, CA, USA) to confirm the specificity of used primers and the accuracy of genotyping.

### Study of gene-gene interactions and risk of DS

All the possible combinations between *RFC1* A80G and *CBS* 844ins68 genotypes were calculated by a 2-by-4 table to measure the excess risk due to the presence of 2 A80G and 844ins68 polymorphisms genotypes in the same woman.

### Ethical considerations

This study was ethically approved by Ahvaz Jundishapur University of Medical Sciences Research Affairs, Ahvaz, Iran (Ethical Code: IR.AJUMS.REC.1397.688) and was experimentally performed in department of Medical Genetics and Cellular and Molecular Research Center. Before starting the project, written informed consent was obtained from all individuals who participated in this project.

### Statistical analysis

Statistical Package for the Social Sciences, version 22.0, SPSS Inc., Chicago, Illinois, USA (SPSS) and Chi-square test were used for data analyses. The Hardy-Weinberg equilibrium (HWE) was tested using allele and genotype frequencies to identify genotyping errors. The odds ratio (OR) and 95% confidence intervals (CIs) in DSM/CM were calculated among 4 frequent genetic inheritance models, including the Allelic model, dominant model, recessive model, and over-dominant model. The genotype can be a major allele homozygote (MM), a heterozygote (Mm) or a minor allele homozygote (mm). The dominant model compares heterozygote (Mm)+ minor allele homozygote (mm) vs. major allele homozygote (MM), recessive model compares the minor allele homozygote (mm) vs. heterozygote (Mm)+ major allele homozygote (MM), over-dominant Model compares heterozygote (Mm) vs. major allele homozygote (mm)+ minor allele homozygote (mm). Probabilistic Model selection was performed using the Akaike information criterion (AIC) value. The best-fit inheritance model with the smallest AIC was considered the inheritance Model. The p-value < 0.05 was considered significant.

## 3. Results

Table I shows the demographic characterization of the study population. In the group DSM, the mean age during the pregnancy of DS was 24.03 ± 5.19 yr, while the CM had a mean age of 22.11 ± 4.89. Serum folate level of both DSM and CM were within the normal range (6–10 ng/mL) and the difference was not significance (p > 0.05). The mean serum concentration of folate in DSM and CM were 7.68.2 ± 1.4 and 9.2 ± 2.83 ngr/mL respectively. In DSM group, each family had only 1 DS child who was not necessarily their first child. Parents of children with DS having normal karyotype and carriers of translocation or iso-chromosomes were excluded from this study.

### Polymorphism *RFC1* A80G was highly detected in DSM 

**Figure 1:**
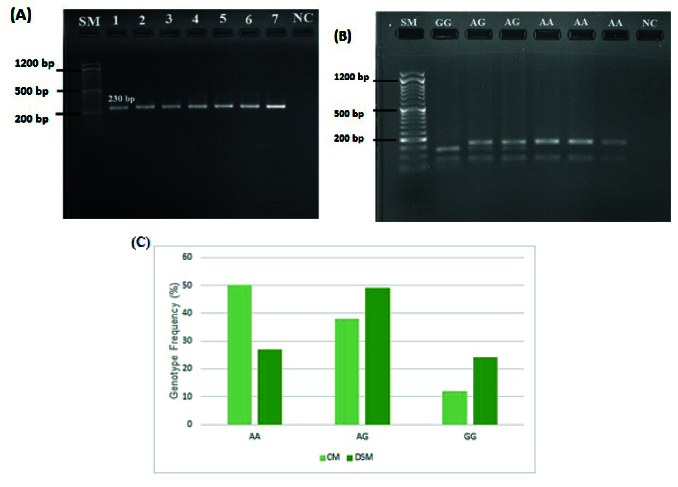
The *RFC1* A80G polymorphism. A) PCR amplification of 230 bp of *RFC1* gene flanking the polymorphism A80G. The SM and NC showed the size marker 100 and negative control, respectively. B) RFLP (CfoI) and agarose gel electrophoresis were applied to distinguish the different *RFC1* A80G polymorphism genotypes. The RFLP pattern showed 68 and 162 bp bands for the AA genotype, bands 37, 68, 125, and 162 bp for the AG genotype, and bands 37, 68, and 125 bp for the GG genotype. The SM and NC indicate the pGEM 100 bp molecular weight marker (Promega) and negative control, respectively. C) Genotype distribution of *RFC1* A80G between CM and DSM.

The genotype of *RCF1* A80G polymorphism was evaluated through an RFLP assay. A 230 bp fragment flanking the A80G polymorphism was amplified during PCR (Figure 1A). Figure 1B shows the RFLP pattern of some participants, illustrating 68 and 162 bp bands for AA genotype, bands 37, 68, 125, and 162 bp for AG genotype, and bands 37, 68, and 125 bp for GG genotype. The genotype frequency of A80G was compared between DSM and CM (Figure 1C). As indicated, a significant difference was observed in genotype frequency of A80G between DSM and CM ( χ 2 = 12.261, p = 0.002) (Table II).

**Figure 2:**
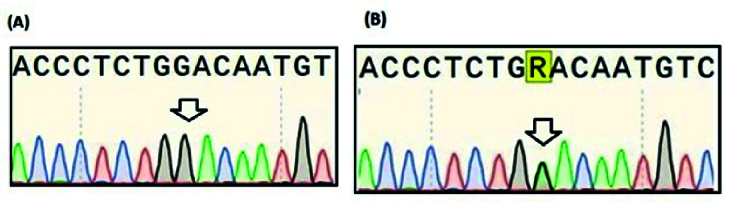
Sanger sequencing confirmed the presence of the 80G variant in people who were determined as Genotype GG A) or Genotype AG B). The arrow shows the position of A80G polymorphism. Sequencing was performed using a forward primer.

Figure 2 shows the results of Sanger sequencing of 2 heterozygous and homozygous participants for A80G. The distribution of all *RFC1* A80G genotypes in our study population was consistent with the Hardy-Weinberg equilibrium ( χ 2 = 0.036, p = 0.82).

Table III compared the frequency of the “A” allele, which was found to be 69% in CM and 51.5% in DSM, While the “G” allele frequency was 31% and 48.5% among CM and DSM, respectively. A significant difference in *RFC1* A80G allelic frequencies was found between DSM and CM ( χ ^2^ = 12.261, p < 0.001), and the frequency of the G allele was significantly higher in DSM than the CM (Table III). Table IV indicates the summary estimates for the OR of *RFC1* A80G for the association between A80G and the risk of DS in allele, dominant, recessive, and over-dominant models. *RFC1* A80G polymorphism was significantly correlated with having DS risk in both dominant and recessive inheritance comparison models (OR = 2.316, p = 0.03 for the recessive model, and OR = 2.704, p = 0.001 in dominant model). The dominant model is the best inheritance model as it yields the strongest association with the smallest AIC (-8.8). The minor G allele was associated with an increased risk of DS (OR = 2.09, 95% CI = 1.39–3.15, p < 0.001) (Table IV).

### Polymorphism *CBS* 844in68 and increased risk of having DS baby in mothers 

**Figure 3:**
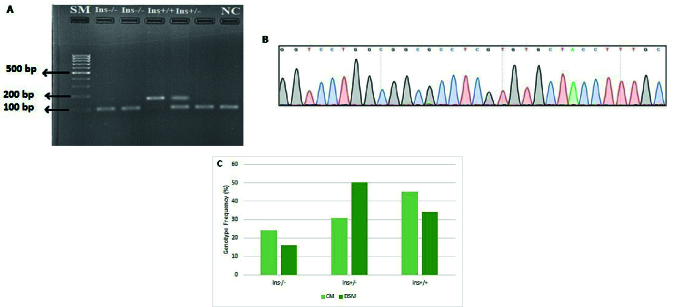
Polymorphism *CBS* 844ins68. A) Polymerase reaction and agarose gel electrophoresis were applied to distinguish the different genotypes of *CBS* 844Ins68 polymorphism. The electrophoretic pattern was a 98 bp fragment for wild-type homozygous genotype (Ins-/-), 2 fragments 98 bp and 166 bp for heterozygous genotype (Ins+/-), and a fragment 166 bp for *CBS* 844ins68 homozygous genotype (Ins+/+). The SM and NC indicate the size marker 100 and negative control, respectively. B) Sanger sequencing confirmed the amplification of 89 bp of *CBS* gene flanking 844ins68 polymorphism. Sequencing was performed using a forward primer. C) Genotype distribution of *CBS* 844ins68 between CM and DSM.

As indicated in the method section, polymorphism *CBS* 844ins68 was genotyped using PCR and electrophoresis. Figure 3A shows the result of agarose gel electrophoresis for some participants. As expected, a 98 bp fragment was observed for the wild-type homozygote genotype (Ins-/-), while 2 fragments of 98 bp and 166 bp were detected in the heterozygous genotype (Ins+/-). The presence of a 166 bp fragment confirmed the homozygous of (Ins+/Ins+).

The true amplification of the *CBS* 844ins68 flanking region was confirmed through the sanger sequencing (Figure 3B). The genotype frequency of 844ins68 was compared between DSM and CM (Figure 3C, Table II). As indicated, a significant difference was observed in genotype frequency of *CBS* 844ins68 between DSM and CM ( χ ^2^ = 7.588, p = 0.0225). The observed genotype frequency for the Ins -/- genotype in DSM was 16% compared to 24% in CM (p = 0.206); Ins+/- genotype frequency was 50% in DSM, and 31% in CM which was statistically significant (p = 0.035), and the Ins+/+ genotype was 34% in DSM compared to 45% in CM (p = 0.216). The genotype distribution of *CBS* 844Ins68 polymorphism significantly differed between CM and DSM ( χ ^2^ = 0.112, p < 0.001). The distribution of all *CBS* genotypes was inconsistent with HWE ( χ ^2^ = 12.261, p = 0.737) (Table II, Figure 3C). The allele frequency of 844ins68 is shown in table III. As indicated, the “Ins +” allele frequency was found to be 0.605 in CM and 0.59 in DSM (p = 0.838). The “ins-” allele frequency was 0.395 and 0.41 among CM and DSM, respectively. No significant difference was observed in SNP allelic frequencies of DSM and CM (p = 0.838) (Table III).

### Gene-gene interaction between *CBS* 844ins68 and *RFC1* A80G polymorphisms

The interaction of *CBS* 844ins68 and *RFC1* A80G in all 200 genotyped DSM and CM was summarized in table IV. As illustrated, a combination of both polymorphisms *RFC1* A80G and *CBS* 844ins68 in a homozygous state (GG/Ins+/+) can significantly increase the risk of DS (OR = 6.667 and p = 0.022).

**Table 1:** Demographic characteristics of the study population

**Study groups**	**Age at conception of DS child**	Serum concentration of folate (ngr/mL)*	**Number of child with DS**
**DSM**	24.03 ± 5.19 (IQR = 8)	7.68.2 ± 1.4 (IQR = 1)	1
**CM**	22.11 ± 4.89 (IQR = 6)	9.2 ± 2.83 (IQR = 2)	0
Data presented as Mean ± SD. CM: Control mother, DS: Down syndrome, DSM: Down syndrome mother, IQR: Interquartile range. The *Reference value of folate, serum in adult women is 5.4–18 ng/mL			

**Table 2:** Genotype frequencies of *RFC1 *A80G and *CBS* 844Ins68 polymorphism in DSM and CM

**Group**		**CM**	**DSM**	P-value*	P-value**
***RFC1*** ** A80G polymorphism**					
	**AA**	50 (50)	27 (27)	< 0.001	
	**AG**	38 (38)	49 (49)	0.238	
	**GG**	12 (12)	24 (24)	0.046	< 0.001
	χ2	12.261				
***CBS*** ** 844 ins68 polymorphism**					
	**ins-/-**	24 (24)	16 (16)	0.206	
	**ins+/-**	31 (31)	50 (50)	0.035	
	**ins+/+**	45 (45)	34 (34)	0.216	0.0225
	χ2	7.588				
Data presented as n (%). Pearson Chi-square. *P-value compared the frequency of each genotype between DSM and CM. **P-value compared the genotype distribution of each polymorphism between DSM and CM while CM: Control mother, DSM: Down syndrome mother					

**Table 3:** Allele frequencies of* RFC1 A80G and CBS* 844Ins68 polymorphism in DSM and CM

**Allele frequencies**		**CM**	**DSM**	χ2	**P-value**
***RFC1*** ** A80G**					
	**A**	138 (69)	103 (51.5)	
	**G**	62 (31)	97 (48.5)	12.787	< 0.001
***CBS*** ** 844ins68**					
	**Ins-**	79 (39.5)	82 (41)	
	**Ins+**	121 (60.5)	118 (59)	0.094	0.838
Data presented as n (%). Pearson Chi-square. CM: Control mother, DSM: Down syndrome mother					

**Table 4:** Interaction between *RFC1* A80G and *CBS *844ins68 and Genotypes in DSM and CM

**Combined genotypes**	**CM**	**DSM**	**P-value**	**OR (95% CI)**
**AA/ Ins -/- (reference group)**	10	3	- -
**GG/Ins +/+**	6	12	0.022	6.667 (1.319–33.693)
**AA/ Ins +/-**	14	17	0.418	1.647 (0.492–5.516)
**AA/Ins +/+**	26	7	0.002	7.429 (2.051–26.911)
**AG/Ins -/-**	8	7	0.251	2.286 (0.558–9.366)
**AG/Ins +/-**	15	27	0.859	1.111 (0.346–3.564)
**AG/Ins +/+**	14	15	0.316	1.867 (0.551–6.329)
**GG/Ins -/-**	5	6	0.515	1.667 (0.358–7.768)
**GG/Ins +/-**	2	6	0.672	0.667 (0.102–4.354)
Data presented as numbers. Logistic regression test. CM: Control mother, DSM: Down syndrome mother, OR: Odds ratio				

## 4. Discussion

This is the first study showing the positive association of *CBS* 844ins68 and *RFC1* A80G polymorphism as a risk factor for DS in Iranian young DS mothers who were residents of southwest Iran.

We found that only the heterozygous state of *CBS* 844ins68 polymorphism was more frequent among DSM than the CMs and half of the DSM were positive for the 844ins variant. The calculated OR was 2.419, which means heterozygous mothers of the 844ins68 variant have around a 2.4-fold increase in the risk of DS than the mothers with other genotypes of 844ins68. The frequency of the 80G allele in DSM (48.5%) is significantly higher than the observed frequency in CM (31%). The OR was calculated as 2.09. We observed that 24% of DSM were homozygous for the 80G variant (GG genotype), and 49% were heterozygous for 80G (AG genotype). In CM, the frequency of GG and AG genotypes were 12% and 38%, respectively.

The gene *RFC1* encodes a transporter of folate into and out of cells. Mutation in the *RFC1* gene can be associated with multiple defects in mice models of birth defect, and supplementation with folic acid can increase the survival of embryos (9). Through HPA RNA-seq normal tissues, a high expression level of *RFC1* was found in the liver, placenta, heart, and whole blood (https://www.ncbi.nlm.nih.gov/gene/6573). *RFC1* has not been directly connected to the increased level of total homocysteine, but it may decrease the availability of folic acid by the developing fetus, resulting in growth problems. Different variants of *RFC1* can influence the concentration of folate metabolism (10). In the latest release of dbSNP (https://www.ncbi.nlm.nih.gov/snp/), 3027 single nucleotide variants have been reported in the *RFC1* gene. However, polymorphism A80G is the most frequent nucleotide change in the *RFC1* gene. The A80G is a missense variant resulting in the substitution of histidine amino acid codon (CAG) to arginine (CGG) and altered concentrations of folate metabolic metabolism products (6). Chango et al. reported that the A80G variant of this gene could increase the total homocysteine level in the homozygous GG state (10). Yet, there appears to be little or no consensus about how to the allele inherited and further analysis is needed. However, it is well documented that the transporter protein derived from the *RFC1* allele 80G is less functional than the protein encoded by the wild-type allele (80A). It has been documented that women who are homozygous of *RFC1* 80G have a 2-fold higher risk of having a baby with a birth defect than those with the 80A variant (11). Totally, 6 studies investigated the association of *RFC1* A80G polymorphism as a maternal genetic risk factor for DS showing the positive association among Italian, French, Romanian, Turkish, Indian, and Iranian mothers as well (Table V).

Similar to Iranian mothers, the Italian and Indian populations showed a positive association between the presence of *RFC1* A80G polymorphism and increased risk of DS. However, no association was observed in a population of Turkish, French, Brazilian, and Romanian. The frequency of the 80G variant in Italian mothers was 49.5%, close to our observed frequency of 48.5% among Iranian mothers (12). This finding was unsurprising as genetic similarity and close relationship between Iranians and Italians based on previously published studies. Iranian-related ancestry was evidenced in Italy's Sardinia and Sicily cities (13, 14). The frequency of the 80G allele was 37.16 among Indian mothers of DS (15). The same story was documented among the Iranian and Indian populations due to the migratory flux between these countries (16). In the case of a population with a lack of association, the unlike results may arise from the difference in their nutritional regime or distinct genetic context.

The *CBS*, a crucial enzyme in the folic acid cycle, is strongly expressed in normal brain tissues and the liver, pancreas, and kidney. The 844ins68 (Allele ID: 231132) is the most common polymorphism of the *CBS* gene, which involves a 68-bp insertion in the coding region of exon8 at position 844. It causes a frameshift, introducing 2 nonsense codons in the same exon. The polymorphism duplicates the intron-exon boundary of exon 8, producing an alternative splicing site (17). In 1995, the *CBS* 844ins68 was first reported in a homocystinuria individuals (18). The current study showed a significant difference in the genotype frequency of *CBS* 844ins68 between DSM and CM. The “Ins+” allele frequency was low (0.605 in CM and 0.59 in DSM. Extensive ethnic and geographic variability was connected with *CBS* 844ins68 polymorphism (19, 20) (Table V).

The frequency of the heterozygous genotype of the 844ins68 allele is around 11.7% in normal North American populations; the Netherlands, Spain, the United States, and Ethiopia have an average prevalence of 11–17%, and the population of China, Czechoslovakia, India, Chile, and Colombia have a low prevalence of 1–10% for heterozygous genotype. The 844ins68 allele has not been reported in Indonesia or Japan (21).

Similar to our result, in a study on the frequency of 844ins68 in Southern Iran, this population showed great similarity with other Caucasian populations, especially in South Italy and North America. In contrast, it varied from East Asian and African populations (22). Although the 844ins68 does not seem to disturb the activity of the *CBS* enzyme, the frequency is increased in people with premature coronary-artery disease (18). The association of *CBS* 844ins68 with DS has been studied in different populations (Table V), but the results were controversial. Fintelman-Rodrigues et al. in a study on the Brazilian people, showed that *CBS* 844Ins68 polymorphism did not correlate with DS pregnancies. While the combined genotype of this polymorphism and other polymorphisms of the folic acid cycle, such as *RFC1* 80G > A, *MTRR* 66A > G, *MTR* 2756A > G, *MTHFR* 1298A > C were associated with the increased risk of having DS babies (23). However, Scala et al. suggested that *CBS* 844Ins68 could not increase the risk of having a baby with DS in Italy (12). Such contradictory results may relate to various factors, including sample size or differences in geographical area and racial background, which should be considered in future studies. We also found that the combination of both *RFC1* GG genotypes with *CBS* 844ins +/+ can increase the risk of having DS babies to 6.667.

One of the valuable points of this study was the evaluation of *RFC1* A80G and *CBS* 844ins68 in 100 young mothers under 35. It has been shown that most DS children have been born to young women, triggering the idea that other risk factors than increased maternal age may be involved in the etiology of DS (24, 25). In 2009, Migliore and Migheli showed that chromosomal nondisjunction and missegregation are multifactorial traits presented by genetics and environment. They found that young mothers of DS children might have a genetic predisposition (like special polymorphisms) to abnormal chromosome segregation in somatic and germinal cells (26). In this study, we selected mothers with a mean maternal age of 24.03 ± 5.19 yr during the pregnancy of DS babies and excluded those over the age of 35 during the pregnancy. In fact, by considering such criteria, we reduced the effect of maternal age on the incidence of DS. Therefore, in mothers of DS children, the syndrome may have arisen from something other than maternal age. Of note, this idea has not been considered in most of the studies that estimated the association of maternal polymorphisms with having DS babies (15, 27).

**Table 5:** The literature review on *CBS *844ins68 and *RFC1 *A80G among different populations

**Polymorphism**		**DSM**	**CM**	**OR**	**P-value**
***CBS*** ** 844ins68**					
	**French, (28)**	119	119	- NS
	**Italian, (12)**	94	264	- NS
	**Brazilian, (23)**	114	110	- NS
	**Turkish, (29)**	47	49	- NS
	**Indian, (30)**	103	100	4.09	< 0.05
	**Iranian, (This study)**	100	100	2.4	0.03
***RFC1*** ** A80G**					
	**Italian, (12)**	94	264	1.48	0.02
	**French, (28)**	119	119	- NS
	**Brazilian, (31)**	67	113	- 0.166
	**Romanian, (25)**	22	42	- NS
	**Turkish, (29)**	47	49	- NS
	**Indian, (15)**	148	130	3.2579	< 0.001
	**Iranian, (This study)**	100	100	2.09	< 0.001
Literature review, CM: Control mother, DSM: Down syndrome mother, NS: Not significant, OR: Odds ratio					

## 5. Conclusion

In conclusion, this study showed that *RFC1* A80G and *CBS* 844Ins68 are 2 polymorphisms of folate metabolism that can be involved in the etiology of DS among Iranian mothers of Khuzestan province.

##  Data availability

Data supporting the findings of this study are available upon reasonable request from the corresponding author.

##  Author contributions 

MTB and NF had full access to all the data in the study and takes responsibility for the integrity of the data and the accuracy of the data analysis. Concept and design: MTB. Acquisition, analysis, or interpretation of data: MTB, NF, PGH and JMA. Drafting of the manuscript: NF, MTB and PGH. Critical revision of the manuscript for important intellectual content: All authors. Statistical analysis: NF and MTB. Supervision: MTB and PGH.

##  Acknowledgments

The authors appreciate the efforts of all colleagues at Ahvaz Jundishapur University of Medical Sciences Research Affairs, Department of Medical Genetics and Cellular and Molecular Research Center for their generous assistance during this project (Grant No: CMRC-9717).

##  Conflict of Interest 

The authors declare that there is no conflict of interest.
